# *Salmonella*-associated Deaths, Sweden, 1997–2003

**DOI:** 10.3201/eid1202.050867

**Published:** 2006-02

**Authors:** Anders Ternhag, Anna Törner, Karl Ekdahl, Johan Giesecke

**Affiliations:** * Swedish Institute for Infectious Disease Control, Solna, Sweden;; † Karolinska Institutet, Stockholm, Sweden

**Keywords:** mortality, population surveillance, *Salmonella* infections, healthy travelers, dispatch

## Abstract

We examined excess deaths after infection with *Salmonella* in a registry-based matched cohort study of 25,060 persons infected abroad and 5,139 infected within Sweden. The domestically infected have an increased standardized mortality ratio, whereas those who acquired *Salmonella* infection abroad had no excess risk of death.

We were interested in studying deaths attributable to *Salmonella* infection. To avoid the problem of misreporting and underreporting when using death certificates, we linked all cases of salmonellosis (with information on country of infection) reported in Sweden to the national civil register on reported deaths during the years 1997–2003. The primary objective was to investigate whether patients with a diagnosis of *Salmonella* infection have a death rate from all causes that differs from that of the general population. If so, the second objective was to determine whether this general mortality rate in previous cases of salmonellosis could be used as a surrogate for *Salmonella*-related death rates. We would in this case expect that any elevated death rate in the *Salmonella* cohort would be highest near time of infection and then gradually diminish and approach the general death rate in the population. Since the patients with domestic cases and patients who contracted the infection abroad may be 2 fundamentally different groups, we analyzed these 2 groups separately.

## The Study

From 1997 through 2003, a total of 30,438 cases were reported to the Swedish Institute for Infectious Disease Control (SMI) of which 25,060 were stated to have been infected abroad. For 239, country of infection was unknown, and they were excluded from the analysis. The median age for the domestically infected salmonellosis patients was 36 years (interquartile range 20–56 years). For persons infected abroad, median age was 40 years (interquartile range 25–56 years).

For general surveillance purposes, SMI receives a file every week from the Swedish National Tax Board with all registered deaths that occurred during the preceding week. This file does not contain any information on cause of death, only the personal identification number and date of death. These data were used to identify *Salmonella*-infected patients who had died after receiving that diagnosis.

For every *Salmonella*-infected patient, whether they died or recovered, follow-up time was counted from the date of onset of illness. Risk time was accumulated until time of death or August 1, 2004. Sex-specific and age group–specific death rates were obtained from Statistics Sweden and were used to calculate the number of expected deaths in the *Salmonella*-infected cohort. The observed number of deaths was divided by the expected number of deaths to produce a standardized mortality ratio (SMR). Poisson regression was used to investigate changes in SMR over time for different strata (expected cases explanatory variable). Exact confidence intervals were calculated, assuming that the number of deaths in each stratum was Poisson distributed.

For the group of persons infected within Sweden (n = 5,139), SMR was increased during every period after onset, falling from 5.6 during the first month to 1.4 after >1 year (Table 1). Within each stratum for time after infection in this group, homogeneity in the results was investigated by calculating separate SMRs for the age groups <14 years, 15–64 years, and >65 years (Table 2). For all time strata, the SMR was approximately equal in all age groups. However, <1 month after infection, SMR = 11.2 (95% confidence interval [CI] 4.1–21.8) for the age group 15–64 years and 4.7 (95% CI 2.6–7.4) for the >65 age group. This difference is not significant because of the small sample, but the assumption that the older age group is responsible for the elevated SMR is highly unlikely.

**Table 1:** Standardized mortality ratios (SMRs) among 30,199 Swedish patients with reported cases of salmonellosis acquired domestically and abroad, 1997–2003*

Time after infection (mo)	Infected domestically (n = 5,139)			Infected abroad (n = 25,060)		
	Obs	Exp	SMR (95% CI)†	Obs	Exp	SMR (95% CI)
<1	21	3.8	5.6 (3.4–8.2)	4	7.0	0.6 (0.2–1.2)
1–3	34	7.2	4.7 (3.3–6.5)	19	14.2	1.3 (0.8–2.0)
4–12	55	30.3	1.8 (1.4–2.3)	36	64.3	0.6 (0.4–0.8)
>12	146	107.3	1.4 (1.1–1.6)	215	341.2	0.6 (0.5–0.7)

*Obs, observed number of deaths; Exp, expected number of deaths; CI, confidence interval.  †p<0.0001.

**Table 2:** Standardized mortality ratios (SMRs) by age group among 5,139 domestically infected *Salmonella* patients, Sweden, 1997–2003*

Time after infection (mo)	Age group (y)	Obs	Exp	SMR (95% CI)
<1	<14	0	0.1	0 (0–59.6)
	15–64	6	0.5	11.2 (4.1–21.8)
	>65	15	3.2	4.7 (2.6–7.4)
1–3	<14	0	0.1	0 (0–30.3)
	15–64	6	1.1	5.6 (2.1–10.9)
	>65	28	6.0	4.7 (3.1–6.5)
4–12	<14	0	0.4	0 (0–7.3)
	15–64	11	4.7	2.4 (1.2–3.9)
	>65	44	25.3	1.7 (1.3–2.3)
>12	<14	1	1.0	1.0 (0.03–3.8)
	15–64	27	16.9	1.6 (1.1–2.2)
	>65	118	89.4	1.3 (1.1–1.6)

*Obs, observed number of deaths; Exp, expected number of deaths; CI, confidence interval.

For persons who had acquired their *Salmonella* infection abroad (n = 25,060), we found no increased deaths compared to the general population; SMR = 0.6 (95% CI 0.2–1.2) <1 month after onset, 1.3 (95% CI 0.8–2.0) after 1–3 months, 0.6 (95% CI 0.4–0.8) after 4–12 months, and again 0.6 (95% CI 0.5–0.7) after >1 year had passed since the acute infection (Table 1). Instead, a significantly lower mortality ratio is evident in this subgroup, compared to that for the Swedish general population, for every period after 3 months have passed since onset of illness. We also calculated SMRs for different age groups separately for the imported salmonellosis cases, but no obvious differences were found between age groups.

Among the isolates that were subtyped, *S*. Enteritidis and *S*. Typhimurium dominated in both of the groups. *S*. Dublin and *S*. Wirchow, which sometimes are believed to be more pathogenic than others, constituted together ≈1% of the isolates among persons infected within Sweden and 2.6% among persons infected abroad.

## Conclusions

Undoubtedly, not all deaths identified by linkage to the civil registration system occurred as a result of *Salmonella* infection, but the finding of a high SMR near time of infection and a steady decrease over time nevertheless indicates that salmonellosis is a contributing factor to the increased risk of death in this group of patients. Studies have shown that of all persons with salmonellosis only a small proportion seek medical care and thus have their case end up in the surveillance statistics (*1*,*2*). However, patients with a severe infection, as well as patients with a travel history before disease onset, are more likely to seek care, receive a diagnosis, and be included in the registry, compared to the average salmonellosis patient (*2*). These 2 patient groups differ greatly with respect to disease severity. Generally, a surveillance system will miss the milder domestic *Salmonella* cases, whereas it will tend to pick up travel-associated cases regardless of severity. A generalization of our results would be the following: SMRs for domestic cases are more representative for severe cases in the population, while the SMRs for travel-associated cases are probably more representative for the milder or moderate cases of salmonellosis.

A Danish study used an approach like ours and found that 3.1% of persons infected with salmonellae were dead within 1 year of infection (*3*). In this study, 0.56% of patients (2.1% with domestic cases and 0.24% with imported cases) were found to have died within the same period. That domestic cases had a more severe prognosis could be 1 explanation for this discrepancy. In Denmark, domestic cases constitute ≈86% of all salmonellosis cases (*4*), whereas in Sweden only ≈17% of cases are reported to be domestic. Death certificates or hospital charts have been used in other studies to measure salmonellosis deaths (*5*–*7*), but none of these studies have thoroughly analyzed the interval from diagnosis to death, used any other population group for comparing death rates, or stratified cases according to presumed country of infection.

In the present analysis, we have not adjusted our results for coexisting illnesses. The assumption that our domestic cases represent a more vulnerable subpopulation is born out by the fact that the SMR for this group remains significantly >1.0 even 1 year after the acute salmonellosis episode.

The most plausible explanation for finding completely different SMRs for persons infected domestically and for those infected abroad is a "healthy traveler effect" (*8*). The least healthy persons in any age group are not those who are traveling abroad.

Future studies on deaths due to salmonellosis should take this healthy traveler effect into consideration and, for domestic cases, also consider the inherent bias of any national surveillance system to include more severe cases.
